# Right paraduodenal hernia: report of two cases and review of literature

**DOI:** 10.1093/gastro/gou076

**Published:** 2014-11-06

**Authors:** Rohit Mehra, Aswini Kumar Pujahari

**Affiliations:** Department of Surgery, Armed Forces Medical College, Pune, Maharashtra, India

**Keywords:** internal hernia, paraduodenal hernia, small bowel obstruction

## Abstract

Paraduodenal hernia (PDH), a rare congenital anomaly, is a type of internal hernia which occurs due to a defect in the reduction and rotation of the midgut. On anatomical and embryological basis, PDH can be broadly divided into right- and Left PDH. Right PDH is rarer than its counterpart. We present two cases of Right PDH. The patientsy presented with a history of recurrent intestinal obstruction since childhood, which was managed conservatively, without a definitive diagnosis. Once they presented to us, a detailed clinical history and a barium meal follow- through clinched the diagnosis of PDH. Intra-operative findings correlated well with the clinical diagnosis. The jejunal loops had herniated through the fossa of Waldeyer. Reduction of hernia contents and excision of the hernia sac was carried out. Post-operatively, the patients are healthy and symptom-free at 4 and 3 years follow-up, respectively. The rarity of this condition and the need for early diagnosis, to prevent the high risk of bowel obstruction and strangulation, makes PDH one of the difficult challenges for the clinicians.

## Introduction

**Figure 1.**
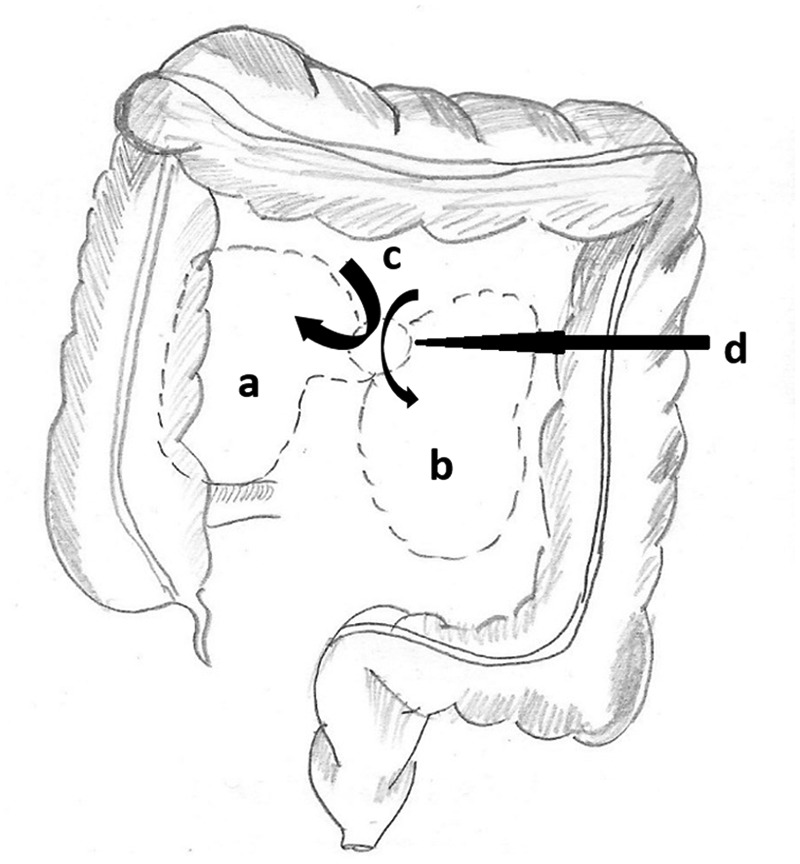
A line diagram depicting the anatomical positions of the colon and fossae formed by fusion of the peritoneal and mesenteric folds: a) the fossa of Waldeyer: where the Right PDH occurs; b) the fossa of Landzert, where the Left PDH occurs; c) the retroduodenum, from where the hernia occurs, and d) the hernial orifice.

Internal hernia is the protusion of viscus through a opening in the peritoneal or mesenteric fold. Paraduodenal hernia (PDH) makes up nearly 50% of all internal hernias. PDH is a rare congenital anomaly that occurs due to an error in the rotation of the midgut, through a normal or abnormal opening in the colonic mesentery [1, 2]. Although often associated with non-specific abdominal symptoms, if missed, PDH can lead to catastrophic outcomes in the form of acute small bowel obstruction, ischaemia and bowel perforation [3]. PDH can be classified as either right- or left-sided, depending on anatomical features and embryological origins. In Right PDH, the viscus herniates into the fossa of Waldeyer. In Left PDH, the herniation occurs in the paraduodenal Landzert’s fossa (Figure 1). Left PDH is three times as common as the right counterpart. Right PDH is seen more in the male sex, with a 3:1 male–female ratio, whereas Left PDH does not have a gender bias [4, 5]. Surgical options, whether open or laparoscopic, aim at hernia reduction and obliteration of the hernial orifice or excision of the sac.

## Case presentation

### Case 1

A 46-year-old gentleman with no known comorbidities presented with two to three episodes of small-volume, non-projectile vomiting, of 3 days duration. There was no abdominal distension and he was able to pass flatus infrequently. He reported a history of similar complaints. The abdomen was soft and non-tender on examination. Bowel sounds were normal. There were no abdominal scars. His haematological and biochemical parameters were within normal limits. Plain X-ray of the abdomen revealed no air fluid levels.

**Figure 2.**
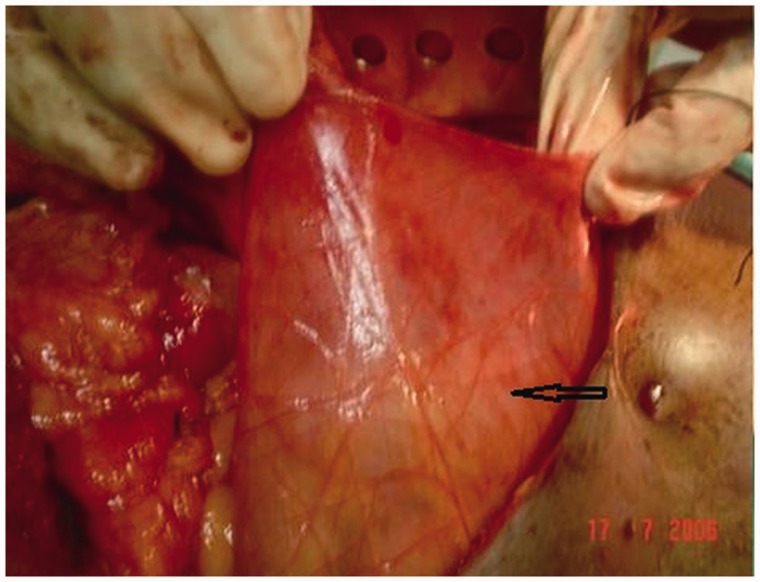
Intra-operative photograph of Case 1, showing a thin hernial sac (marked by an arrow) enclosing the small bowel.

The patient was managed conservatively with intravenous fluids. He showed signs of clinical improvement in the first 72 hours, but the abdominal pain and vomiting restarted thereafter. He was therefore taken up for a surgical intervention. The peritoneal cavity was approached through a midline laparotomy. Intra-operatively, there was a thin hernial sac covering the small bowel (Figure 2). The hernial sac was opened laterally, to preserve the duodenum and superior mesenteric vessels, and excised. This repositioned the small bowel in the normal anatomical position. The hernial orifice was generously widened to prevent any future bowel incarceration. The inferior mesenteric vein, which was to the left of the hernial sac, was preserved. The post-operative period was uneventful and the patient has been asymptomatic in the 4 years following surgery.

### Case 2

A 23-year-old gentleman with no known comorbidities presented with complaints of colicky pain in the epigastric and umbilical region, distension and three episodes of bilious, non-projectile, small-volume vomiting over two days. He also had associated complaints of infrequent passage of flatus for the past day. There was no history of abdominal surgery or trauma to the abdomen. He gave a history of similar complaints of recurrent abdominal pain of variable intensity since childhood, which required hospitalization. During each of his previous eight hospital admissions, he had been managed conservatively, without a definite diagnosis.

**Figure 3.**
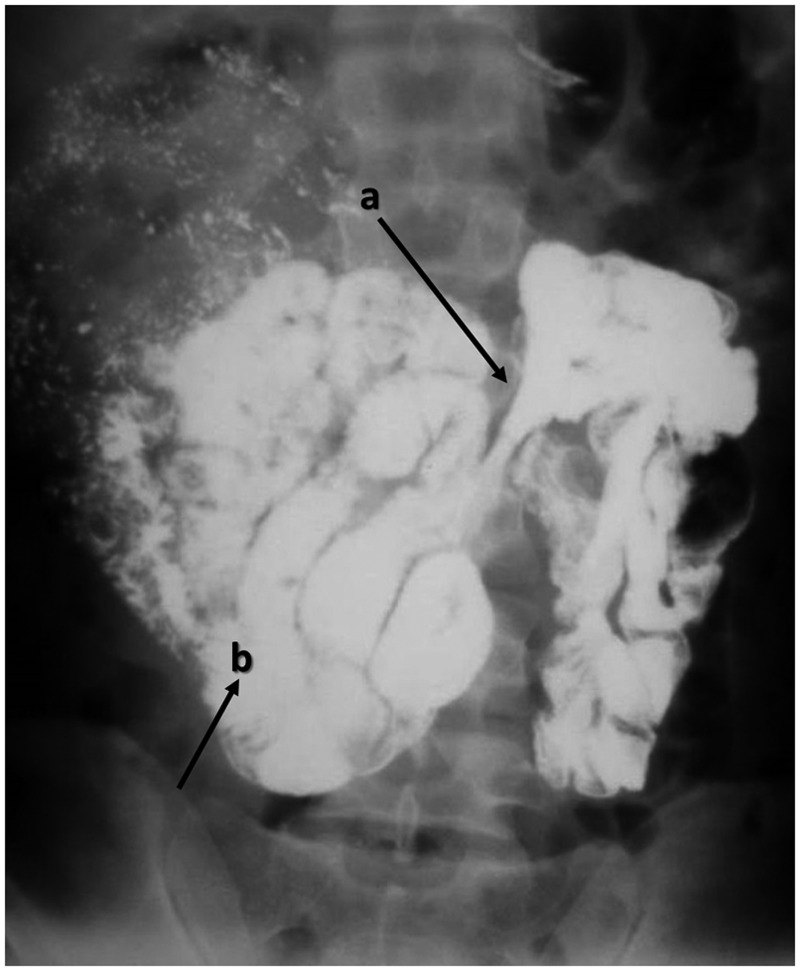
A plain erect abdominal X-ray of Case 2, showing right paraduodenal hernia. The opening of the fossae of Waldeyer is marked by arrow a). small bowel loops are clustered to the right of the midline (marked by arrow b), in a well circumscribed hernial sac.

On physical examination, the pulse and blood pressure was found with-in normal limits. The abdomen was non-tender but mildly distended—more so towards the right, with exaggerated bowel sounds. Digital rectal examination revealed a collapsed rectum. All the haematological and biochemical parameters were within normal limits. There was a single dilated small-bowel loop on plain X-ray of the abdomen. The patient was managed conservatively with intravenous fluids and he improved in 72 hours. In view of his repeated hospitalization for bowel obstruction and in the absence of an abdominal scar, a rare, congenital cause was thought possible and a barium meal and follow-up was recommended. This demonstrated oval grouping of the jejunal loops, encapsulated within the hernial sac, predominantly to the right of the midline (Figure 3). Upper gastrointestinal endoscopy was normal. X-ray of the chest and Mantoux test were negative for tuberculosis.

**Figure 4.**
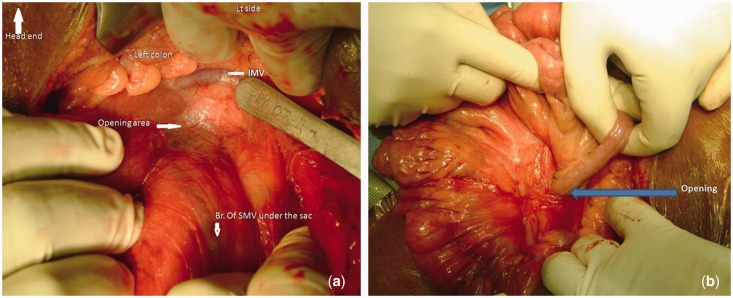
Intra-operative photograph of Case 2: a) showing the hernial sac and its relationship with the superior mesenteric and inferior mesenteric veins; b) showing delivery of incarcerated small bowel loops from the hernial orifice (marked by the arrow).

On elective diagnostic laparoscopy, the colon was in its normal anatomical location, barring a small loop of small bowel at the duodeno-jejunal flexure; the rest of the small bowel was covered by a hernial sac. The mouth of the hernial sac was located to the right of the inferior mesenteric vein (Figure 4A). After delivery of few loops of jejunum through the opening, a significant resistance was felt. Fearing injury during the delivery of the intestine, conversion to open laparotomy was made. The small bowel was pulled out of the hernial opening (Figure 4B). The hernial sac was cut open and excised from the duodenal jejunal flexure as far as the ileocaecal junction, preserving the superior mesenteric vessels. The hernial orifice was generously widened, as in Case 1, to prevent any future bowel incarceration. The entire small bowel was normal, except an area of adhesion to the posterior part of the membrane, which was the cause of resistance during laparoscopy. The patient had an uneventful post-operative period and has been asymptomatic for the last 3 years.

## Discussion

Neubauer, in 1786, is credited with the first description of PDH in the history of medical science [2]. He ascribed it to faults in peritoneal development. Nearly a century later, Treitz described the peritoneal folds and fossae, through which the hernia retroperitonalis develops. Over these years, several theories on the development of PDH were formulated and discarded. The two theories that have stood the test of time are:

- *Moynihan’s Theory*: he attributed PDH to a condition known as ‘physiological adhesions’, which arise at the time of return of the bowel back to the abdomen and fusion of the common dorsal mesentery with the posterior abdominal wall. This leads to the formation of fusion folds and fossae (Nine such fossae were described by him). Gradual enlargement of such fossae often leads to development of PDH. The two most important fossae implicated are the fossa of Landzert (for Left PDH) and fossa of Waldeyer (for Right PDH), as depicted in Figure 1.
- *Andrews' Theory*: Andrews respected Moynihan’s concept of fusion folds and fossae but doubted the gradual enlargement of the fossae. He ascribed the condition to the developmental fusion defects of peritoneum, which incarcerated the small bowel beneath the developing colon [6, 7].

**Figure 5.**
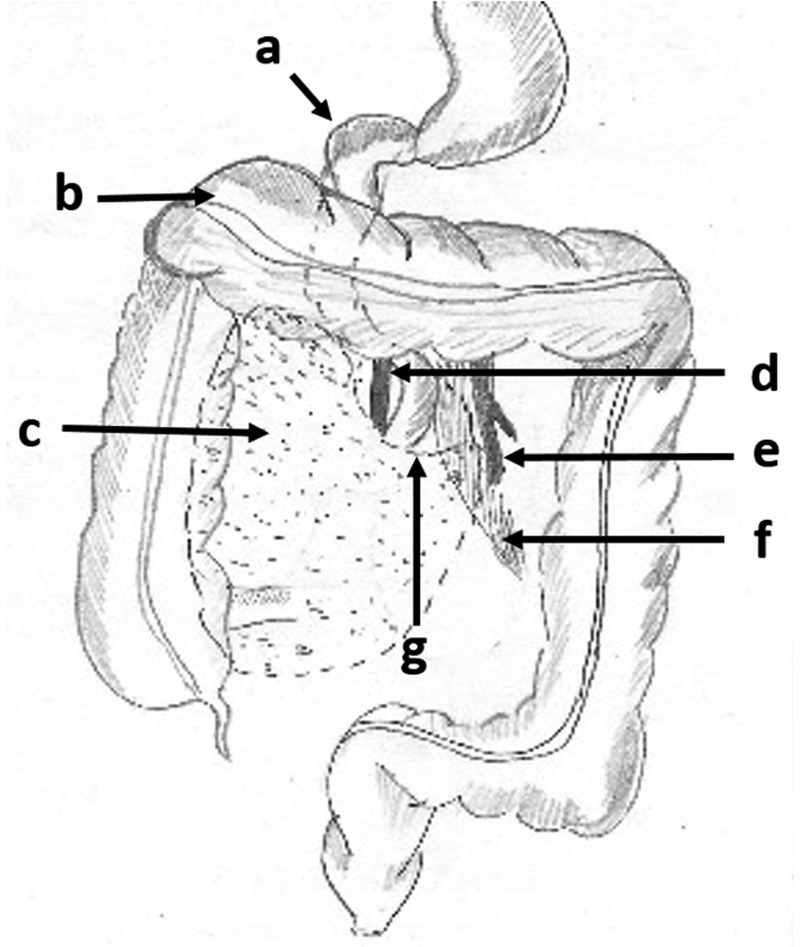
A line diagram showing the anatomical landmarks in a right paraduodenal hernia: a) the first part of the duodenum; b) the transverse colon; c) the hernial sac forming the fossae of Waldeyer; d) the superior mesenteric vein; e) the inferior mesenteric vein; f) the mesentery of the herniated bowel and g) the hernial orifice.

In cases of Right PDH, the counter-clockwise rotation of the midgut during embryological development is arrested on the right side. The small bowel becomes trapped in a hernial sac formed by the peritoneum, behind the colonic mesentery, and the caecum and ascending colon rotate anteriorly, as shown in Figure 5 [8].

Despite the fact that nearly half of all internal hernias are paraduodenal hernias, there are few cases reported in the medical literature, and these mostly as isolated case reports. Although internal hernias account for only 1% of all cases of intestinal obstruction, nearly 50% of all patients with PDH develop obstruction [2]. Patients present with a history of recurrent pain in the abdomen and ill-defined complaints, which often leads to misdiagnosis; history of partial or complete intestinal obstruction might also be present. However, small bowel contrast radiography is the lynchpin of pre-operative diagnosis [2, 9]. PDH has unique characteristics under radiography, which can help in formulating a definitive diagnosis: these have not changed since 1921, when Kummer first described them as “total absence of small intestine in the true pelvis in the upright position; the small bowel is confined in a smooth, sharply circumscribed mass"—the ‘classical empty abdominal sign' [10]. In Right PDH, the small bowel is trapped behind the mesocolon of the ascending and right transverse colon. The superior mesenteric artery is usually found on the free edge of the hernial sac, as in our cases. Kummer’s description was illustrated in a precise, text-book manner in the radiological findings of our second case. To differentiate the Right- from Left PDH, Exner suggested that if the circumscribed bowel mass lies to the left of the midline, it is Left PDH, and *vice-versa* [11]. The advent of computed tomography (CT) has eased the brain-storming sessions previously required to arrive at the diagnosis [12]. In our first case, the typical presentation and the clinical suspicion clinched the diagnosis while, in the second case, the small bowel contrast study obviated the need for a CT scan.

The treatment approach for PDH is based on the principle of hernia reduction, plus either repair of the defect or widening of the hernial orifice [2]. As the superior mesenteric vessels lie in close proximity to the hernial orifice, any attempt to open the sac at the hernial orifice should be discouraged. Two principle approaches for the surgical management of PDH have been widely publicized. In the first, the sac is opened wide laterally, after identifying the duodenum and avoiding injury to the superior mesenteric vessels, in order to release the incarcerated small bowel into the peritoneal cavity. Once the sac wall is excised, the pouch effect towards the pelvis vanishes, and the anatomical location of the bowel is maintained. This is the approach that was followed in both of our cases. The second approach is division of the lateral attachments of the right colon and mobilizing it to the left, opening the hernia sac wide and replacing the pre- and post-arterial segments of the intestine in the positions they would normally occupy at the end of the first stage of rotation, during embryonic development [8].

There are few case reports covering the herniation of the small bowel through the lesser sac, leading to incarceration, with presentations akin to PDH. Surgical widening of the hernial orifice and resection of the strangulated bowel, if any, with primary anastomosis, has been reported in the literature [13]. With the laparoscope changing the face of abdominal surgery, the above-mentioned approaches can be tried laparoscopically, but the dictum of *primum non nocere* should always supervene.

In conclusion, PDH remains elusive in its diagnosis. A reliable anatomical knowledge of the peritoneal and mesenteric folds and, in cases with chronic recurrent abdominal pain with partial obstruction, a high degree of suspicion, are of paramount importance. The technological advantages of radiology should be sought early in such cases, so that a prompt surgical intervention prevents the high morbidity and mortality associated with PDH.

*Conflict of interest statement:* none declared.
